# Blood-based immunophenotyping of T cell profiles in patients with neurodegenerative disorders

**DOI:** 10.1186/s13195-026-02144-y

**Published:** 2026-07-16

**Authors:** Frederika Malichova, Peter Swann, Stacey L. Kigar, Natalia Savinykh Yarkoni, Julia Goddard, Kei Onn Lai, Leonidas Chouliaras, Ajenthan Surendranathan, Lorinda Turner, George Savulich, Richard Bevan-Jones, Nicholas J. Ashton, Kaj Blennow, Henrik Zetterberg, Edward Needham, Joanne Jones, William A. McEwan, James B. Rowe, John T. O’Brien, Maura Malpetti

**Affiliations:** 1 Department of Clinical Neurosciences, University of Cambridge, Cambridge University Hospitals NHS Trust, Cambridge, UK; 2 Department of Psychiatry, University of Cambridge, Cambridge, UK; 3 Department of Medicine, University Cambridge, Cambridge, UK; 4 UK Dementia Research Institute at University of Cambridge, Cambridge, CB2 0XY UK; 5 Specialty Dementia and Frailty Service, Essex Partnership University NHS Foundation Trust, Epping, UK; 6 University College London Hospitals NHS Foundation Trust, London, UK; 7 Department of Psychiatry and Neurochemistry, University of Gothenburg, Gothenburg, Sweden; 8 Banner Alzheimer’s Institute and University of Arizona, Phoenix, AZ USA; 9 Banner Sun Health Research Institute, Sun City, AZ 85351 USA; 10 Clinical Neurochemistry Laboratory, Sahlgrenska University Hospital, Mölndal, S-431 80 Sweden; 11 Department of Neurodegenerative Disease, UCL Institute of Neurology, Queen Square, London, UK; 12 UK Dementia Research Institute at UCL, London, UK; 13 Hong Kong Center for Neurodegenerative Diseases, InnoHK, Hong Kong China; 14 Wisconsin Alzheimer’s Disease Research Center, School of Medicine and Public Health, University of Wisconsin, University of Wisconsin-Madison, Madison, WI USA; 15 Medical Research Council Cognition and Brain Sciences Unit, Cambridge, UK; 16 Department of Clinical Neurosciences, University of Cambridge, Herchel Smith Building, Forvie Site Robinson Way, Cambridge Biomedical Campus Cambridge, Cambridge, CB2 0SZ UK

## Abstract

**Background:**

There is increasing evidence for the role of central and peripheral inflammation across neurodegenerative disorders, with animal models and post-mortem studies identifying T-cell infiltration in the brain associated with pathology and neurodegeneration. Peripheral T-cell changes have been measured in Alzheimer’s disease (AD), dementia with Lewy bodies (DLB), frontotemporal dementia (FTD) and progressive supranuclear palsy (PSP). This study examines a unique cohort of blood-based T-cell profiles across a range of neurodegenerative dementias including AD, DLB, FTD, corticobasal syndrome (CBS), PSP, and aged-matched healthy controls. Then it also explores their associations with dementia-relevant plasma biomarkers and clinical outcomes.

**Methods:**

Freshly prepared peripheral blood mononuclear cells (PBMCs) from 174 participants (AD = 20, DLB = 24, FTD = 19, CBS = 18, PSP = 58, controls = 35) were studied using a flow-cytometry panel designed to analyse major T-cell subpopulations, including memory and T-helper subtypes. Neurodegeneration-relevant biomarkers (p-tau217, p-tau231, GFAP, NfL, and A-beta42/40) were measured in plasma samples. T-cell populations were compared between groups and in association with biomarkers, and principal components analysis (PCA) was used to identify T-cell profiles and their association with dementia-relevant biomarkers in diagnostic classification and survival prediction.

**Results:**

There was a significant reduction in the fraction of CD3+ cells in patients with DLB compared to other diagnostic groups, and an increase in relative Th1/17-like cell levels in patients with FTD compared to controls. This increase in Th1/17-like cells correlated with NfL and GFAP plasma levels in patients with FTD. PCA identified five components primarily representing CD4+ memory cell population subsets. After sex and age adjustments, component 4 marked by effector memory types including Th2-like, Th-like1 and Th1/17-like cells was a significant predictor of FTD, however was not as accurate as plasma NfL. Higher scores in specific T-cell components (1 and 3) were associated with reduced mortality across all diseases, with component 3 remaining a significant predictor even when controlling for traditional neurodegenerative biomarkers like NfL and p-tau217.

**Conclusions:**

This study provides evidence that T-cell dysregulation is not unified in patients with neurodegenerative diseases. We observe different involvement across different dementia types establishing adaptive immunity as a key contributor to disease heterogeneity. However, although plasma biomarkers such as NfL and p-tau217 exhibit superior diagnostic accuracy for clinical classification, peripheral T-cell signature were associated with survival outcomes across diagnostic groups, highlighting their promise for prognostic applications and disease monitoring. The characterisation of T-cell populations across neurodegenerative conditions may inform target development and patient stratification for new interventional trials.

**Supplementary Information:**

The online version contains supplementary material available at https://doi.org/10.1186/s13195-026-02144-y.

## Background

The primary roles of the immune system are healing and defence against pathogens and harmful agents including bacteria, viruses, toxins, or damaged cells [1]. Broadly, it can be classified into two branches: the innate and the adaptive immune system. The innate immune system is the first line of defence against pathogens and consists of cells including monocytes and neutrophils. The adaptive immune system is antigen-specific and has long-lived memory cells, allowing rapid recognition of previously encountered antigens. The adaptive immune system consists of two major cell populations, the B and T-cells [2].

Neuroinflammation is established as a hallmark across neurodegenerative diseases [3]. Microglial activation, as measured by positron emission tomography (PET) of the translocator protein (TSPO), is dementias [4–7]. Increasingly associated with disease progression and clinical severity in patients with neurodegenerative, studies are exploring the role of peripheral immune system [8–11], including changes in myeloid cells across neurodegenerative diseases [10]. Systemic inflammation has been shown to exacerbate neuropathology in animal models of neurodegeneration and is associated with disease progression in clinical cohorts [12]. This may be mediated by T-cell migration across the blood-brain/blood-cerebrospinal fluid barrier [13–15]. T-cell infiltration promoting astrocytosis, microgliosis and neurodegeneration has been reported in mouse models of tauopathy [16, 17] and the presence of T-cells in human post-mortem brain tissue from patients with Alzheimer’s disease (AD), frontotemporal lobar degeneration (FTLD), and dementia with Lewy bodies (DLB) [18–22]. The role of T-cells in neurodegeneration is not clear, with some studies finding associations with neuronal loss [23], whilst others highlight a potential protective role of specific T-cell subsets [24].

Although the peripheral adaptive immune system, and specifically T-cell populations, plays a role in neurodegeneration, further studies are needed, particularly comparing AD and non-AD dementias, to identify whether T-cell changes are disease-specific or transdiagnostic across neurodegeneration. Characterisation of these cells could lead to clinically relevant blood-based biomarkers to support target identification and patient stratification for immune-targeting therapies. Conflicting evidence on the proportions of CD4 + and CD8 + T-cell proportions has been reported in AD [25–31] and related animal models [32]. There are studies of peripheral T-cell immunophenotyping, predominantly in AD (reviewed in [33]) reporting, for example, the changes in T-cell subtypes such as CD4 + Th17, Th1 and Th2 like cells increase in AD patients [30] as well as their association with cognition [28] and its accumulation prior dementia onset [34]. However, very few have looked at this across multiple neurodegenerative diagnoses [11, 25–29, 35]. A single cohort including multiple neurodegenerative diseases using the same immunophenotyping methodology across all patients, in conjunction with plasma neurodegenerative biomarkers and survival, remains scarce.

This study, therefore, aims to characterise T-cell profiles in blood from people with neurodegenerative diseases causing dementia, including AD, DLB, frontotemporal dementia (FTD), corticobasal syndrome (CBS) and progressive supranuclear palsy (PSP). Using flow cytometry, we immunophenotyped freshly prepared peripheral blood mononuclear cells (PBMCs) to characterise and quantify T-cell sub-populations. We tested three hypotheses: firstly, that there are differences in the fraction of major T-cell populations (CD3+, CD4+, CD8+, memory subsets and T-helper lineages) across neurodegenerative disorders that correlate with other neurodegenerative biomarkers, secondly, that T-cell profiles provide useful information to classify neurodegenerative diagnosis, and finally, that T-cell profiles are associated with survival.

## Methods

### Participants

Patients were recruited from clinics for cognitive and movement disorders at the Cambridge University Hospitals NHS Trust as well as collaborating local psychiatry and neurology services. We included participants with AD (comprising people who met criteria for Mild Cognitive Impairment (MCI) [36] with evidence of AD pathology or standardised criteria for AD [37] (*n* = 20), CBS [38] (*n* = 18), DLB [39] (*n* = 24), FTD (*n* = 19; 13 behavioural variant FTD (bvFTD) and 6 primary progressive aphasia (PPA)) [40, 41], and probable or possible PSP (*n* = 58, predominantly Richardson’s syndrome) [42]. Age-matched healthy volunteers (*n* = 35) were included as the control group, with MMSE > 26/30, absence of memory symptoms, no signs of dementia, or other significant medical illnesses [43]. Exclusion criteria for both patient and healthy control cohorts included major concurrent psychiatric illness, other severe physical illness, or a history of other significant neurological illness. For participants with mental capacity, all gave written informed consent according to the Declaration of Helsinki. For participants who did not have the mental capacity to consent, the consultee process was followed. The study protocol was approved by the NIHR National Research Ethic Service Committee and East of England (Cambridge Central; REC Ref: 13/EE/0104; 07/Q0102/3).

Participants underwent a standardized clinical assessment with the Addenbrooke’s Cognitive Examination [44] (ACE-R), which represents a global measure of cognition scored out of 100. The ACE-R can be subdivided into five domains: Attention and Orientation (18/100), Memory (26/100), Fluency (14/100), Language (26/100), and Visuospatial abilities (16/100). Survival data were collected for all participants up to and including the 27th November 2024 (the census date).

### Blood collection and flow cytometry

At baseline, 18 ml of blood was drawn into EDTA blood tubes and analyzed at the NIHR Cambridge BRC Cell Phenotyping Hub. Wherever possible, samples were processed within 2 h (over 98% of samples). Blood was layered onto sterile Ficoll (Cytiva, Cat#: 17144003) for peripheral blood mononuclear cell isolation by a technician blind to group status. Two aliquots containing ~ 1 × 10^6 PBMCs were labelled using live-dead stains and antibodies for CD3, CD4, CD8, CXCR3, CD45RA, CCR7, and CCR6. At the end of staining, cells were washed, and the data were acquired on live (i.e., non-fixed) cells with a BD LSR Fortessa instrument. Cell classifications were determined via manual gating in FlowJo (BD) by individuals blind to the participant group according to standards recommended for the Human Immunology Project [45] using an agreed gating strategy (Supplementary Fig. 1). We also performed an analysis of the control group across multiple years which revealed no significant changes or batch effects in CD4 and CD8 counts (Supplementary Fig. 2). For clarity of communication, we defined CD3+ cells as T-cells, CCR7+ CD45RA+ cells as naïve, CCR7+ CD45RA- cells as central memory (CM), CCR7-CD45RA- cells as effector memory (EM), and CCR7-CD45RA+ cells as terminally differentiated effector memory cells re-expressing CD45RA (EMRA/TEMRA). In the CD4⁺ T-cell subset, we also defined CXCR3 + CCR6- CD4+T-cells as Th-like1 cells, CXCR3-CCR6- CD4+ T-cells as Th2-like cells, CXCR3-CCR6+ CD4+ T -cells as Th17-like cells and CXCR3+ CCR6+CD4+ T-cells as Th1/17-like cells (Th1/17-like cells were not defined as a numerical sum of Th1-like and Th17-like subsets), including only memory populations in these subsets (by removing naïve cells defined as CCR7 + CD45+) and recognising that these are enriched rather than pure populations of Th subtypes. Summary can be found in the Supplementary Table 3. The median number of live events recorded for each participant was 387,208, and the median for the CD4+ memory total was 65,628. All subpopulations had a median number of cells greater than 100. Whilst the fewest cells were in CD4+ TEMRA subpopulation, all but three of the participants (98%) had greater than 100 CD4+ TEMRA cells measured. The median cell numbers in each gate are shown in Supplementary Table 1. Cell population frequencies were calculated relative to live singlet events within the PBMC morphological gate or relative to the parent cell population, as specified for each result. Although the focus of this study was T-cell populations, in order to ensure differences were not driven by large changes in other immune cell populations we also performed flow cytometry in the majority of samples (*n* = 165) with a broad panel including antibodies to CD45, CD16, CD56, and CD19, alongside CD3, CD4, and CD8 (Supplementary Fig. 3).

### Plasma biomarkers relevant to neurodegenerative diseases

In a sub-cohort of 124 participants (AD = 18, CBS = 13, DLB = 22, FTD = 14, PSP = 31, controls = 26), plasma samples stored at -70 °C were used for further analyses at the Clinical Neurochemistry Laboratory in Mölndal (Sweden). Plasma samples were thawed on wet ice, centrifuged at 500×g for 5 min at 4 °C. Calibrators (neat) and samples (plasma: 1:4 dilution) were measured in duplicates. The plasma assays performed were the Quanterix Simoa Human Neurology 4-Plex E assay (measuring Aβ40, Aβ42, GFAP and NfL, Quanterix, Billerica, MA), the p-tau217 ALZpath assay measuring p-tau217 of the human tau protein associated with AD, and p-tau231 with an in-house Simoa assay as previously described [46, 47]. Samples were analysed at the same time using the same batch of reagents. A four-parameter logistic curve fit data reduction method was used to generate a calibration curve. Two control samples of known concentration of the protein of interest (high-control and low-control) were included as quality control. Intra-assay coefficients of variation were below 10%. The mean plasma biomarker concentrations for each group are shown in Supplementary Tables 2 and the demographic differences between those with and those without plasma biomarkers are shown in Supplementary Table 3.

### Statistical analysis

Statistical analyses were carried out in R for frequentist tests and JASP for Bayesian tests. Differences between sex were compared with the Chi-squared test. Group differences on continuous variables were compared Kruskal-Wallis Chi-squared test, followed by Dunn’s post-hoc test with Benjamin-Hochberg corrections for multiple comparisons. Where there were differences in demographic covariates, linear models were used with the relevant covariates with the T-cell population fraction as the outcome and group as a predictor. Bayesian ANOVA was used in JASP using ranks, and Bayes Factor compared to the null model reported. A Bayes Factor (BF10) between 1 and 3 is regarded as ‘mild’, between 3 and 10 as ‘moderate’, between 10 and 30 as ‘strong’ and above 30 as ‘very strong’ evidence for H1 over H0.

Principal components analysis (PCA) was performed using the *prcomp* R function, selecting components with an adjusted eigenvalue > 1 from Horn’s parallel analysis, and applying a varimax rotation to the retained components. Individual component scores were extracted and used as variables in multinomial and then logistic regression. Models were compared with the likelihood ratio test. Correlations were performed with Spearman’s rank correlation coefficient. Cox proportional hazards models were fitted using time from blood draw to death as the outcome, with participants still alive at the census date (27th November 2024) treated as right-censored. The date of blood sampling was used as the time origin, as it represents an objectively and uniformly recorded time point at which T-cell profiles would realistically be obtained in a clinical setting. T-cell component scores were included as predictors, with age, sex, diagnostic group, and ACE-R score as covariates. In this analysis, we included 136 participants with dementia (53 deceased, 83 alive) and did not include controls. Cox proportional hazard models were performed in R with the time between blood draw and death (or census date if the patient was still alive) as the outcome, and T-cell component scores as predictors.

## Results

### Demographics and baseline cognition

One hundred and thirty-nine participants with neurodegenerative dementias were recruited and thirty-five cognitively unimpaired controls (Table 1). Overall, there was no significant difference in age, but there were differences in sex (DLB group proportionally more male than CBS group) As expected, all dementia groups had lower cognitive scores, as measured by the ACE-R, compared to controls *p* < 0.001, and AD and FTD lower than PSP, t=-3.46, *p* = 0.009, t=-2.94, *p* = 0.043 respectively, post-hoc Dunn tests).

**Table 1:** Demographics and baseline cognition

	Control *N* = 35^1^	Dementia(all) *N* = 139	AD *N* = 20^1^	CBS *N* = 18^1^	FTD *N* = 19^1^	PSP *N* = 58^1^	DLB *N* = 24^1^	All dementia vs. control^2^	Individual group differences^3^
Age	69.0 (6.4)	71.3 (7.5)	71.0 (6.0)	69.3 (8.9)	69.3 (7.6)	71.7 (7.1)	73.4 (8.0)	W = 1966, *p* = 0.080	H = 8.7, *p* = 0.12
Sex								X^2^=0.42, *p* = 0.5	X^2^ =14.3, *p* = 0.014
F	13 (37%)	60 (43%)	8 (40%)	12 (67%)	6 (32%)	30 (52%)	4 (17%)		
M	22 (63%)	79 (57%)	12 (60%)	6 (33%)	13 (68%)	28 (48%)	20 (83%)		
ACER	95.9 (3.4)	74.3 (17.2)	66.2 (14.5)	75.1 (17.7)	67.9 (23.8)	79.6 (14.1)	73.3 (15.9)	W = 4389, *p* < 0.001	H = 75.6, *p* < 0.001

*M* male, *F* female

^1^: Mean (SD); n (%)

^2^: Wilcoxon rank sum test; Pearson’s Chi-squared test

^3^: Kruskal-Wallis rank sum test; Pearson’s Chi-squared test

### Group differences in major T-cell populations

**Fig. 1:**
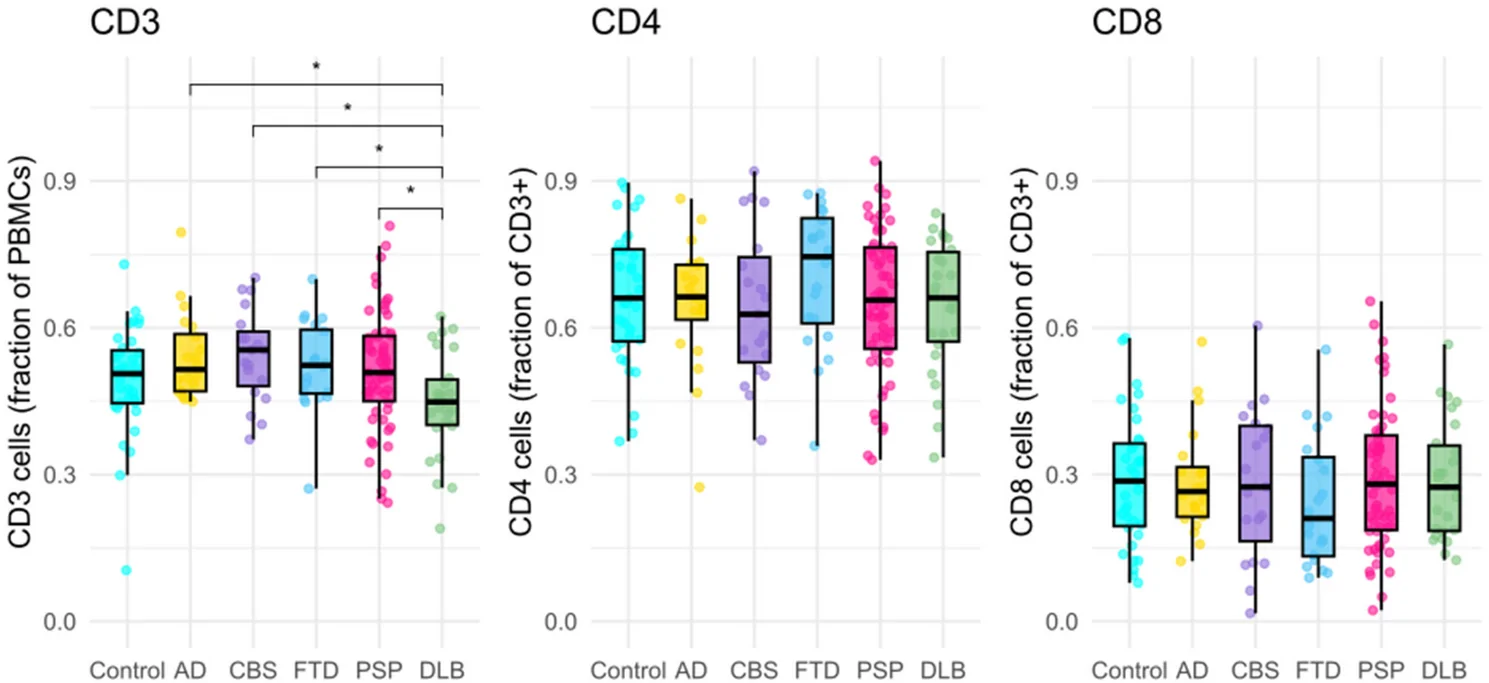
Group differences in CD3+, CD4+ and CD8+ T-cell populations. Box and dot plots display CD3+ (left), CD4+ (middle), and CD8+ (right) cell fractions between groups. Each bar represents a different group (AD- Alzheimer’s disease, CBS- corticobasal syndrome, FTD- frontotemporal dementia, PSP- progressive supranuclear palsy, DLB- dementia with Lewy bodies), with dots representing individuals within that group. Asterisks indicate significance at *p* < 0.05 with FDR correction from Dunn’s post-hoc test with Benjamin-Hochberg correction following a significant Kruskal-Wallis test

There were significant group differences in CD3 + cells, as a fraction of live PBMCs (H = 13.0, *p* = 0.023, BF_10_=1.25, supplementary results 1.1), with participants in the DLB group having significantly lower CD3 + levels than all other disease groups (Dunn’s post-hoc test with Benjamin-Hochberg corrections, DLB vs. AD: Z =-2.79, pFDR = 0.033, BF_10_=14.79; DLB vs. FTD: Z=-2.47, pFDR = 0.014, BF_10_=3.58; DLB vs. PSP: Z=-2.72, pFDR = 0.033, BF_10_=2.98; DLB vs. CBS: Z=-3.02, pFDR = 0.031, BF_10_=13.93, supplementary results 1.2), with moderate to very strong evidence from Bayesian ANOVAs (see Fig. 1A). However, the identified difference in CD3 + cells between patients with DLB and controls was not significant (Z=-1.94, uncorrected *p* = 0.052, corrected *p* = 0.16) with the Bayes-Factor (BF_10_=0.91). These results remained similar when controlling for age and sex, in a linear model with CD3 + cell fraction as the main outcome and diagnostic group as the main predictor (DLB vs. AD: *p* = 0.012; DLB vs. FTD: *p* = 0.090, DLB vs. PSP: *p* = 0.043; DLB vs. CBS: *p* = 0.034, supplementary results 1.3). This could represent either a decrease in CD3 + cell levels, or an increase in other cell types leading to the proportional decrease. Using a separate broad immunophenotyping panel, we showed there were no major shifts in monocytes, natural killer cells, natural killer T-cells, or B-cells that would explain this result, suggesting it does reflect a reduction in CD3 + cells in DLB **(**supplementary results 1.4&1.5).

There were no significant group differences in CD4+ cells (H = 3.0, *p* = 0.69, BF_10_ =0.034, supplementary results 1.6&7) or CD8+ cells (H = 2.37, *p* = 0.80, BF_10_=0.026, supplementary results 1.8&9) as a fraction of CD3+ cells (Kruskal-Wallis test, Bayesian ANOVA) (Fig. 1). Likewise, there were no significant group differences in the CD4+/CD8 + ratio (H = 2.43, *p* = 0.79, BF_10_ =0.067, supplementary results 1.10).

### Group differences in T-cell subpopulations

**Fig. 2:**
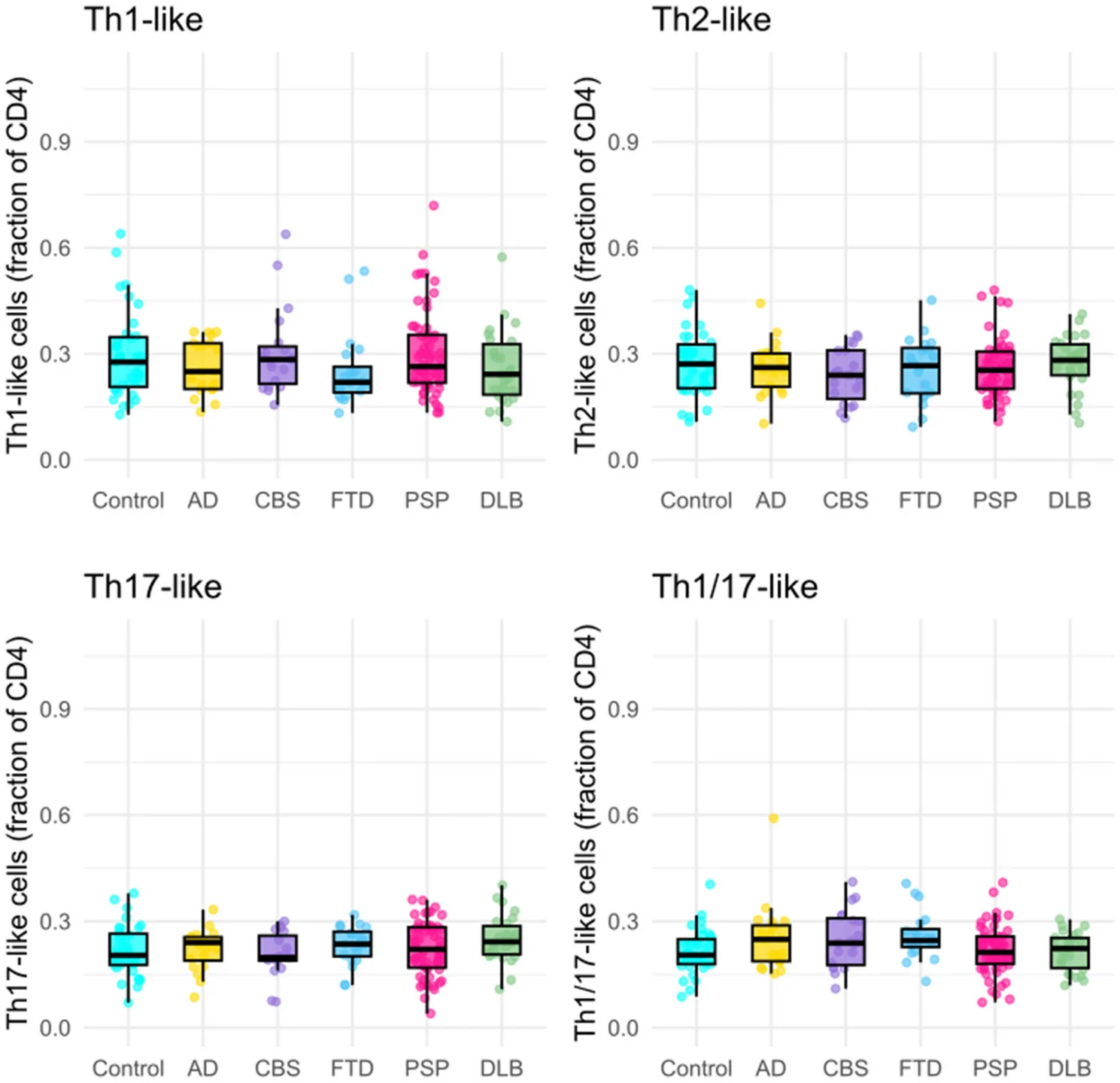
Group differences in Th1-like, Th2-like, Th17-like, and Th1/17-like cells. Box and dot plots displaying the Th populations as a fraction of total memory cells. Each bar represents a different group, with dots representing individuals within that group (AD- Alzheimer’s disease, CBS- corticobasal syndrome, FTD- frontotemporal dementia, PSP- progressive supranuclear palsy, DLB- dementia with Lewy bodies)

Between groups, as a fraction of total memory CD4+ T-cells, there were no significant differences in Th1-like (H = 5.76, *p* = 0.33, BF_10_ =0.10), Th2-like (H = 3.03, *p* = 0.70, BF_10_ =0.04), Th17-like (H = 5.60, *p* = 0.35, BF_10_ =0.08), or Th1/17-like cells (H = 8.76, *p* = 0.12, BF_10_ =0.64, Kruskal-Wallis, Bayesian ANOVA, supplementary results 1.11–1.17) However, when controlling for age and sex the linear model with Th1/17-like cells as outcome variable and diagnostic group as main predictor identified significant differences in patients FTD (t = 2.28, *p* = 0.024) as compared to controls (Fig. 2, Supplementary results 1.18).

There were no group differences in naïve (H = 413 *p* = 0.53, BF_10_ =0.06), CM (H = 7.88 *p* = 0.16, BF_10_ =1.05), EM (H = 4.95 *p* = 0.42, BF_10_ =0.09), or TEMRA (H = 9.80 *p* = 0.08, BF_10_ =0.14) cells as a fraction of overall CD4+ cells (Kruskal-Wallis test, Bayesian ANOVA, supplementary results 1.19–1.26), and this was unchanged when including age and sex in the linear models. Regarding CD8+ cells, there was similarly no overall group differences in naïve (H = 6.57 *p* = 0.25, BF_10_ =0.15), CM (H = 9.89 *p* = 0.08, BF_10_ =0.17), EM (H = 8.44 *p* = 0.13, BF_10_ =0.16), or TEMRA (H = 7.55 *p* = 0.18, BF_10_ =0.27) cells as a fraction of overall CD8+ cells (Kruskal-Wallis test, Bayesian ANOVA) (supplementary results 1.27–1.34). However, the linear models including age and sex as covariates identified a reduction in naïve CD8+ cells in PSP (t=-2.12, *p* = 0.036).

### Association of T-cell populations with plasma dementia-relevant biomarkers

Next, we explored the relationship between T-cell populations and dementia-related plasma biomarkers with Spearman’s partial correlations, correcting for age and sex. We focused on the cell populations identified as showing different fractions between disease groups and controls (CD3+ cells in DLB; naïve CD4 cells in PSP, and Th1/17-like cells in FTD).

The fraction of CD3+ cells in patients with DLB did not significantly correlate with Abeta42/40 ratio (rho=-0.26, *p* = 0.26), p-tau217 (rho=-0.31, *p* = 0.19), p-tau231 (rho=-0.11, *p* = 0.64), GFAP (rho = 0.27, *p* = 0.24) or NfL (rho = 0.05, *p* = 0.84) in partial correlations controlling for age and sex (Supplementary Fig. 4).

Similarly, the fraction of naïve CD8+cells in PSP did not correlate with Abeta42/40 ratio (rho = 0.3, *p* = 0.339), p-tau217 (rho = 0.2, *p* = 0.13), p-tau231 (rho = 0.16, *p* = 0.27), GFAP (rho=-0.02, *p* = 0.91) or NfL (*p*=-0.02, *p* = 0.19) (Supplementary Fig. 5).

**Fig. 3:**
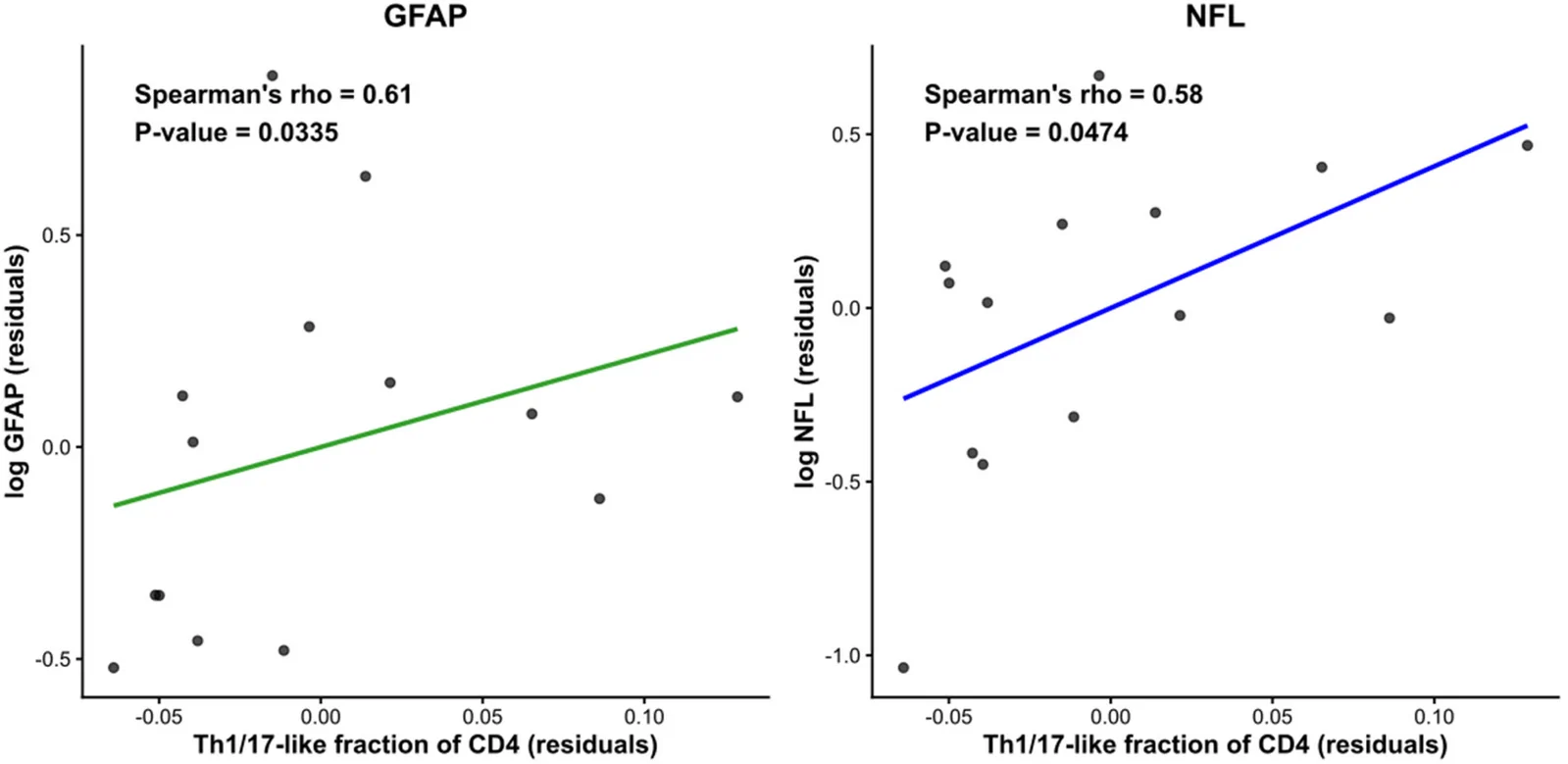
Associations of Th1/17-like memory cell fractions as a percent of total memory CD4+ cells with NfL (left) and GFAP (right) in patients with FTD. Plots show the residuals of log(NfL) or log(GFAP) and Th1/17-like cell fraction from a linear model including age and sex (as significantly associated covariates)

In FTD, whilst there were no significant correlations between Th1/17-like cells and p-tau217 (rho = 0.34, p-0.28) or p-tau231 (rho = 0.30, *p* = 0.34) (Supplementary Fig. 6), there were strong and significant correlations with GFAP (rho = 0.61, *p* = 0.03) and NfL (rho = 0.58, *p* = 0.047) (Fig. 3).

### PCA identifies T-cell profiles across neurodegenerative diagnosis

**Fig. 4:**
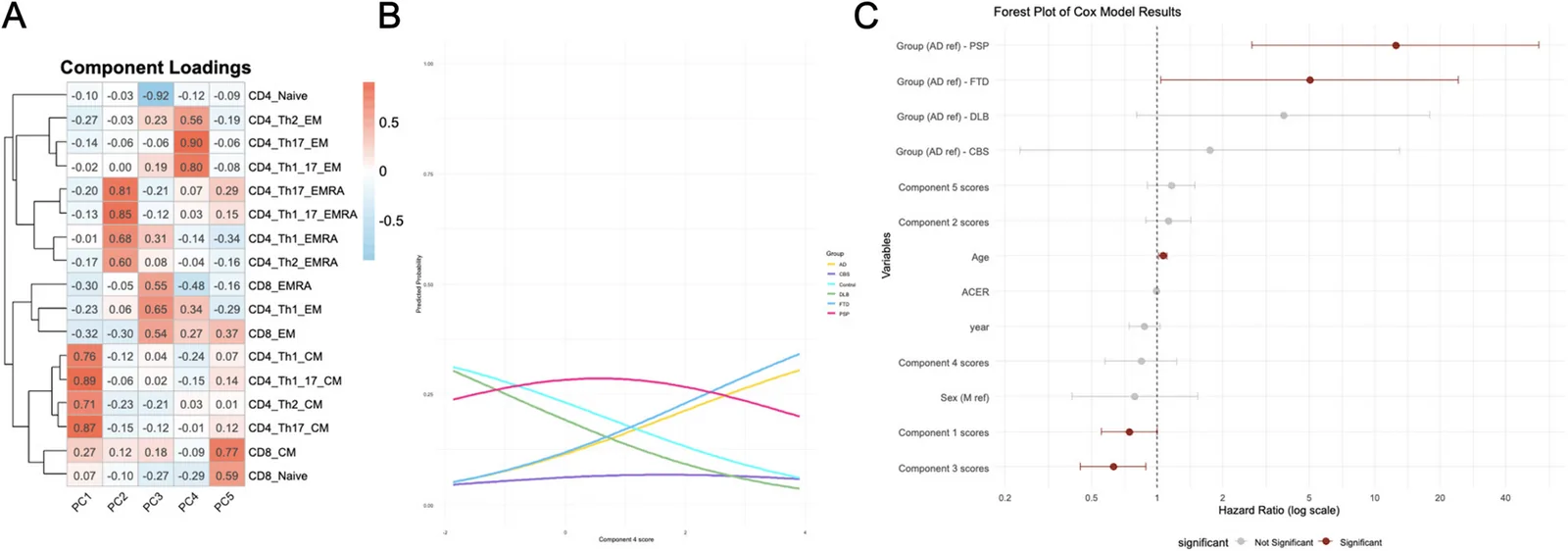
T-cell profiles across diagnosis are predictive of survival. **A**: Heatmap showing the loadings of four principal components identified by PCA. Each row represents a T-cell subpopulation and each column a component, with the colour showing the loading of each T-cell population in that component. **B**: Marginal effects plot showing the predicted probability of each diagnostic group controlling for age and sex. This shows that increased component 3 scores are associated with an increased predicted probability of AD and FTD compared to other diagnoses. **C**: Forrest plot showing the results of the Cox proportional hazards model including the principal component scores representing T-cell profiles, with diagnosis, age, sex, and ACE-R as covariates. This shows that higher component 1&3 scores are associated with decreased mortality, whilst age and a diagnosis of progressive supranuclear palsy (PSP) or frontotemporal dementia (FTD) (compared to the reference group, Alzheimer’s disease (AD)) were associated with increased mortality

Finally, we included 17 subpopulations, both CD4+ and CD8+, as a fraction of total live CD3+ cells in a PCA (Supplementary Table 4). The overall MSA was 0.50 and Bartlett’s Test of Sphericity was significant (*p* < 0.0001), suggesting the data were adequate for PCA. Results of Horn’s parallel analysis suggested including five components, which in total explained 69% of the data (supplementary Fig. 7). The variable loadings of components 2, 4, and 5 were predominantly negative so both variable loadings for these components were multiplied by -1 for interpretability. The component loadings reflected the hierarchical gating, with each component broadly representing a CD4+ memory cell phenotype (component 1 = CM, component 2 = TEMRA, component 3 = naïve, and component 4 = EM), with component 3 also including effector and EMRA CD8+ cells, component 4 also predominantly weighted on the CCR6+ populations, i.e., Th1/17-like and Th17-like cells, and component 5 capturing naïve and CM CD8+ cells (Fig. 4A). Next, we used multinomial regression to see if scaled individual scores for these components were significant predictors of diagnostic groups, with Wald Chi squared test to assess significance of individual predictors. Using diagnostic group as the outcome, with age and sex as covariates, we identified component 4 (representing CCR6+ EM CD4+ populations) as a significant predictor of FTD (odds ratio = 1.84, CI = 1.01–3.38, *p* = 0.048). AD had a similar effect size, but this result was not significant (odds ratio = 1.81, CI = 0.96—3.39, *p* = 0.064) (Supplementary results 1.35).

### T-cell profiles in diagnostic classification

To test the usefulness of this T-cell profiles in FTD for differential diagnoses, we compared the component scores in logistic regression models with (i) FTD vs. controls, and then (ii) FTD vs. other diseases. We considered age and sex as covariates and as a basic model, compared to a model also including individual scores for each component. Component 4 scores improved classification of FTD compared to controls (AUC = 0.66, *p* = 0.036, AIC = 75.6) but not other diagnosis (AUC = 0.66, *p* = 0.16, AIC = 117.8). The diagnostic performance of component 4 was not as strong as plasma NfL levels (AUC = 0.98 vs. controls, AIC = 18.9, AUC = 0.73 vs. other diagnosis, AIC = 79.3). The addition of component 4 to NfL did not significantly improve the model (AIC = 20.6, *p* = 0.58). These findings underscore that, in this cohort, plasma NfL captures most of the diagnostic signal, and that T-cell component scores should be viewed as complementary marker of the disease progression rather than stand-alone diagnostic tools. Details of the models are including in supplementary results 1.36–1.46, and receiver operating curves (ROC) for these models are shown in Supplementary Fig. 8.

### T-cell profiles predict survival

Finally, we assessed the association of the T-cell components with mortality using Cox proportional hazards models controlling for age, sex, diagnostic group, and total ACE-R score.

Components 2, 4 and 5 did not show significant prognostic effects. Increased component 1 scores (representing CD4+ CM cells) were associated with reduced mortality (HR = 0.74, *p* = 0.047). Increased component 3 scores, representing lower fractions of CD4+ naïve cells and higher fractions of Th1-like EM, CD8+ EM and CD8+ EMRA cells, were associated with reduced mortality (HR = 0.63, *p* = 0.009). Compared to the reference group of AD, PSP (HR = 12.44, *p* = 0.001) and FTD (HR = 5.01, *p* = 0.044) were associated with increased mortality, as was age (HR = 1.06, *p* = 0.014, supplementary results 1.47). To assess how this compared to other neurodegenerative biomarkers, we tested component 3 scores in a model with NfL and p-tau217, where it remained a significant predictor of mortality (HR = 0.57, *p* = 0.009), whilst component 1 scores did not (HR = 0.87, *p* = 0.48) (Fig. 4C, supplementary results 1.48).

## Discussion

In this study, a large cohort comprising 174 patients with neurodegenerative diseases and age-matched healthy controls was investigated to characterize T-cell immune profiles using comprehensive and data-driven methodologies on peripheral blood mononuclear cells. Leveraging the uniqueness of this cohort and advanced techniques for immune phenotyping, we identified marked differences in peripheral T-cell subpopulations between diagnostic groups, which were associated with clinical diagnosis, survival outcomes, and established pathology biomarkers. These findings provide robust support for the mounting evidence that T-cell immunity is intricately involved not only in the pathogenesis of AD but also in non-AD dementias, underscoring a critical role for adaptive immune alterations across diverse neurodegenerative syndromes.

First, we identified group differences in CD3+ cell counting, but not in CD4+ and CD8+ total cell counts. Specifically, patients with DLB showed a lower fraction of CD3+ cell (relative to live PBMCs) than the rest of the diagnostic groups and controls. Notably, while some prior studies in patients with DLB have reported a reduction in blood CD4+ T-cell counts [11], the decrease in overall CD3+ T-cell fraction, which reflects reduced total T-cell levels, may indicate more global T-cell alterations in DLB that extend beyond the helper subset. Previous evidence suggests that T-cells are recruited into the brain in DLB [19], with CD4+ T-cells identified in the brain and CSF in patients with DLB [20]. This migration may explain the observed peripheral depletion, as T-cells re-localise from the blood to the central nervous system in response to neuroinflammatory signals. These findings align with prior reports that patients with DLB have abnormal T-cell trafficking and increased evidence of central immune activation [19], supporting the hypothesis that peripheral immunological shifts mirror central immune processes. The reduction in circulating CD3+ T-cells in DLB adds further support to suggestions that altered immune homeostasis is a disease-relevant process in Lewy body disorders.

Previous research in animals showed Th1-like and Th17-like cells accelerated memory impairments and neuroinflammation in animal models of AD [48], human studies showed an increase in CCR6+ and T-cell memory populations in AD [30, 49, 50], PBMCs stimulated with amyloid beta shift towards a Th17-like phenotype in AD compared to controls [51], and a meta-analysis has also described increases in these Th1-like and Th17-like-associated cytokines in AD, including TNF-alpha and IL-6 [52]. Expanding on previous research focused on single neurodegenerative diseases compared to controls, we investigated the group differences in T sub-cell populations, after age and sex adjustments.

Whilst we did not find any significant differences in patients with AD, we found significant differences between FTD and controls in the Th1/17 subpopulation. There have been fewer studies of the T-cell profiles in FTD [53], however increases in Th1-like and Th17-like cells have been associated with disease severity in clinical ALS [54, 55] and animal models [56]. Recent work has shown peripheral cytokines that are functionally linked to Th1-like and Th17-like responses (including TNF-alpha, IL-12, IL17A, and IL-6) are also associated with lower survival rates in patients with FTD and related disorders [4]. Using these results for further exploratory associations showed that in patients with FTD Th1/17-like cell counts correlated with plasma NfL and GFAP in FTD, suggesting an association between peripheral T-cells, astrocyte activation and neurodegeneration. Few studies have explored the links between T-cells and dementia biomarkers, with one finding of a blunted Th17-like response associated with cognition, NfL, and GFAP in women with MCI [57] and another finding associations with CD8+ T-EMRA cells, NfL and GFAP in a community cohort of ageing and mild dementia [58]. Secondly, we found significant reduction of naive CD8+ subpopulation between patients with PSP and controls. This finding is consistent with previous investigation, where reduced percentage of CD8+ naïve cells was reported between patients with PSP and controls in peripheral blood mononuclear cells alongside with a hypothesis of its conversion to a differentiated CD8+ memory subtype, supported by an increase of these cells in patients with PSP [59].

Next, our data-driven approach identified 5 components. Upon investigating their relevance to clinical symptoms, one of the components showed improved classification of FTD compared to controls. Effector memory CD4+ T-cells have been found in higher fractions in patients with AD than in healthy controls and linked with cognitive deficits [35, 60, 61], suggesting their potential role in the disease course and dementia-related neuroinflammation. The pathogenic role of EM CD4+ cells in accelerating memory impairment, amyloid deposition, microglia activation, and neuroinflammation in AD has further been demonstrated in animal models [48]. Mechanistically, effector memory CD4+ cells are producers of pro-inflammatory cytokines and have the capacity to cross the blood–brain barrier during inflammatory states, interacting with microglia and amplifying neuroinflammatory cascades. This mechanism has been implicated not only in AD, but this works potentially expands this into other neurodegenerative diseases such as FTD, suggesting a shared vulnerability driven by peripheral immune dysfunction.

Further, we investigated how the components relate to mortality of the patients. We found that increased component 1 and component 3 scores are associated with reduced mortality.

Component 1 represented central memory CD4+ T-cells and component 3 represents higher fractions of Th1 EM, CD8+ EM and CD8+ EMRA cells and lower fractions of naive CD4+ cells. This study does not explore the mechanisms for these associations or identify casual linkage, however, there are several hypotheses that may explain this finding. Enhanced immune cell migration into tissues, including the brain, would reduce their fraction in the periphery, and T-cell infiltration into the brain has been associated with tau pathology and neurodegeneration [23]. Alternatively, T-cell activation has been proposed to be beneficial in neurodegeneration. Studies have explored enhancing the peripheral immune response in AD [62], and T-cell exhaustion and association with loss of function may be a therapeutic target [63]. Finally, lack of effective peripheral immune populations may also put individuals at higher risk of infection [57], a leading cause of mortality in dementia [64, 65].

Despite some commonalities across studies, there are wide variations in the literature on T-cells in patients with dementia [33]. For example, in our cohort we did not identify a reduction in CD4+ cells, in contrast to previous reports in patients with AD cases [26], or the associated shift in the CD4+/CD8+ ratio, which has been previously described in blood of PSP patients [59]. Other studies, however, have also not replicated these findings [25], identifying shifts towards more later differentiated CD4 T-cells with senescence markers [66], or increases in CD4 T-EMRA cells in AD compared to controls [26]. The inconsistent findings between studies may reflect differences in cohorts [26], the panel design [66] and antibody choice [35], with some studies on freshly collected PBMCs and others on those stored in liquid nitrogen [26]. Studies with larger cohorts based on clinical and biomarker criteria across dementia groups will allow a greater understanding of the relationship between peripheral immune changes and disease progression, and stratification by disease stage or co-pathology (such as AD co-pathology in CBS or DLB).

The diagnosis-specific T-cell shifts observed here point to distinct peripheral immune responses across neurodegenerative diseases, consistent with growing evidence that adaptive immunity is shaped by the underlying pathological substrate. Several mechanisms may contribute: antigen-driven T-cell activation by CNS-derived proteins with concomitant redistribution to the brain [67, 68]; redistribution of lymphocytes to tertiary lymphoid organs such as the meninges [69, 70] or other sites of peripheral inflammation; disease-specific T-cell loss as a consequence of, accelerated immune ageing, including altered rates of thymic involution or lymph node deterioration; and unaccounted for between-group differences in disease course, due to our currently limited ability to define early and late stages of disease, which in animal models affects T-cell trafficking to the brain [71, 72]. These mechanisms associated with aging and chronic neurodegeneration are not mutually exclusive and may operate to different degrees across disease subtypes. Genetic factors are also likely modulators of these responses; APOE ε4 carriage, for example, has been associated with reduced IL-7/IL-7R signalling and lower CD28 expression in effector memory T-cells, consistent with impaired T-cell survival and maintenance correlating with hippocampal atrophy [73], and represents an important covariate for future studies. Resolving the directionality of peripheral T-cell shifts - whether they contribute to neurodegeneration, arise secondarily to CNS pathology, or reflect broader systemic immune dysregulation - will require functional immune data, longitudinal sampling, and integration with paired CNS measures, which are key priorities for future work. However, the number of T-cells in the CNS parenchyma may be too small to account quantitatively for measurable reductions in peripheral T-cell frequencies. Another possible interpretation is that the proportional decrease in circulating CD3+ cells in DLB reflects relative expansion of other leukocyte populations - most plausibly monocytes and NK cells - rather than absolute peripheral T-cell depletion. This is consistent with findings from our companion innate immune study in an overlapping cohort, in which patients with Lewy body disease showed a particularly distinct peripheral innate immune profile, characterised by shifts in NK cell subtypes and TREM2+ monocyte dynamics but not consistent with the broader TruCount panel we have shown there are no major shifts in other cell populations [10].

This study has several limitations. Patients were recruited and included in groups defined on the basis of clinical diagnostic criteria, and amyloid biomarker positivity confirmation for patients with AD. This limits biological precision and likely contributes to diagnostic and pathophysiological overlap between groups. Future studies would benefit from applying similar methods in cohorts of patients with in depth biomarker-confirmed diagnoses and/or with pathology-confirmed diagnoses. This study characterised immunophenotypes at a single timepoint in individual participants. Immune processes in AD, similarly to other diseases, have been proposed to peak early in disease [9], and functional genomics have linked an initial proinflammatory response to tau pathology and to later immunosuppression [74]. Future studies with longitudinal biosampling are required to identify how these immune changes progress over individual disease courses. Our study focused on peripheral blood mononuclear cells and did not measure infiltration of T-cells into the brain or CSF, which could reveal complementary information and evidence for the role of T-cells in neurodegeneration. Similarly, PET imaging was available only in a limited sub-cohort of participants. Combining peripheral measures of T-cell profiles with CSF or PET imaging for neuroinflammation (e.g. TSPO PET), and ultimately pathological measures of inflammation will lead to a more comprehensive understanding of the immune landscape across neurodegeneration.

Importantly, our immunophenotyping panel relied on surface markers rather than intracellular staining to classify the major T-cell subpopulations. For example, in our study Th2-like cells were defined by lack of CXCR3 and CCR6 without CCR4, while Th17-like cells were defined as CXCR3-CCR6+ and may contain a very small population of Th2-like cells which would be CCR10+. The lack of CD56 in our panel meant that natural killer T-cells (NKTs) were not separated from CD8+ T-cells. Recent advancement in flow cytometry has enabled the characterisation of larger panels improving the classification of subpopulations, and with high parameter spectral flow cytometry leading to the detection of novel immune subpopulations in neurodegeneration [28]. Future efforts to replicate these findings in an independent cohort and using larger cell phenotyping panels across a range of neurodegenerative dementias, integrating single cell RNA sequencing and T-cell receptor repertoire analysis [75], will be important to gain further insight in T-cell profiles in dementias. Whilst, this study focuses solely on T-cells, analysis of the myeloid immune compartment of a substantial proportion of this cohort has been characterised in a companion paper [10]. Notably, we refrained from pairing these data with circulating cytokine measurements; although such integration would strengthen functional interpretation, basal plasma cytokine levels typically display high inter-individual variability and often sit near or below the detection limits of standard assays.

Future studies combining PBMC phenotyping with high-sensitivity multiplexed plasma cytokine profiling, as we have begun to implement in recent work using mass cytometry and NULISA-based assays [76], will be important to establish functional links between the T-cell shifts reported here and downstream inflammatory signalling. In addition, immunophenotyping was performed on fresh samples on the day of collection, which prevents cryopreservation-related cell death and changes in expression markers associated with freezing and thawing [77]. However, this could introduce batch effects. Future studies could include internal controls to measure this with greater precision. Finally, we did not measure several confounders that could influence T-cell phenotypes such as APOE [73] and for viral infection, notably herpes simplex virus type I (HSV-1) [78] and cytomegalovirus (CMV) [58]. Although time from disease onset to death is clinically intuitive, onset is often difficult to define reliably in neurodegenerative disorders, so we used the date of blood sampling as an objectively recorded and clinically meaningful time origin at which prognostic T-cell profiles could realistically be obtained.

Overall, this study using uniform methodology within a single cohort combining T-cell immunophenotyping with longitudinal survival follow-up across multiple neurodegenerative diagnostic groups provides evidence of significant alterations in peripheral T-cell subpopulations in neurodegenerative diseases. These alternations correlate with distinct clinical diagnoses and plasma biomarkers of neurodegeneration. While plasma NfL and p-tau217 demonstrated superior diagnostic accuracy, the immune profile characterized by higher fractions of CD4+ naïve cells and lower fractions of Th1-like effector memory cells was strongly associated with survival outcomes. This suggests that T-cell immunophenotyping may offer valuable prognostic information and serve as a potential biomarker to monitor treatment response in immunomodulatory trials. With the advent of novel T-cell-targeting therapies emerging for neurodegenerative diseases, an improved understanding of disease-specific T-cell profiles and the development of robust immune biomarkers will be crucial for patient stratification and optimizing clinical trial design.

## Supplementary Information

Supplementary Material 1.

## Data Availability

Anonymized processed data can be shared upon request with the corresponding author. Raw data may also be requested but are likely to be subject to a data transfer agreement with restrictions required to comply with participant consent and data protection regulations.
