# Supplementary material for: Blood-based immunophenotyping of T cell profiles in patients with neurodegenerative disorders
Source: Alzheimers Res Ther. 2026 Jul 16;18:230. doi: 10.1186/s13195-026-02144-y (PMC13656490; doi:10.1186/s13195-026-02144-y)
Supplement: Supplementary file 1 [file 13195_2026_2144_MOESM1_ESM.docx]

# 1. Supplementary results

## 1.1. CD3 by Group

| **Chi_Squared** | **df** | **P_Value** |
| --- | --- | --- |
| 12.995 | 5 | 0.0234 |

## 1.2. CD3 Dunn Post-Hoc

**Output**

.y. group1 group2	n1	n2 statistic	p p.adj p.adj.signif

1 CD3_flow DLB	Control	26	35	1.94 0.0524 0.157 ns

2 CD3_flow DLB	AD	26	20	2.79 0.00526 0.0331 *

3 CD3_flow DLB	CBS	26	19	3.02 0.00251 0.0331 *

4 CD3_flow DLB	FTD	26	20	2.47 0.0136 0.0510 ns

5 CD3_flow DLB	PSP	26	58	2.72 0.00661 0.0331 *

6 CD3_flow Control AD	35	20	1.17 0.242	0.519 ns

7 CD3_flow Control CBS	35	19	1.44 0.150	0.376 ns

8 CD3_flow Control FTD	35	20	0.827 0.408	0.681 ns

9 CD3_flow Control PSP	35	58	0.648 0.517	0.705 ns

10 CD3_flow AD	CBS	20	19	0.256 0.798	0.798 ns

11 CD3_flow AD	FTD	20	20	-0.304 0.761	0.798 ns

12 CD3_flow AD	PSP	20	58	-0.729 0.466	0.699 ns

13 CD3_flow CBS	FTD	19	20	-0.556 0.578	0.723 ns

14 CD3_flow CBS	PSP	19	58	-1.03 0.305	0.572 ns

15 CD3_flow FTD	PSP	20	58	-0.359 0.720	0.798 ns

## 1.3. CD3 Linear Model

| **Term** | **Estimate** | **Std_Error** | **T_Value** | **P_Value** |
| --- | --- | --- | --- | --- |
| (Intercept) | 0.690 | 0.085 | 8.083 | 0.0000 |
| groupControl | 0.030 | 0.028 | 1.050 | 0.2950 |
| groupAD | 0.081 | 0.032 | 2.531 | 0.0123 |
| groupCBS | 0.072 | 0.034 | 2.133 | 0.0344 |
| groupFTD | 0.055 | 0.032 | 1.707 | 0.0897 |
| groupPSP | 0.053 | 0.026 | 2.033 | 0.0437 |
| Age | -0.003 | 0.001 | -2.722 | 0.0072 |
| SexM | -0.027 | 0.017 | -1.613 | 0.1085 |

## 1.4. Kruskal-Wallis Tests

Table 1. Kruskal-Wallis Test Summary

| **Cell Type** | **Chi_Sq** | **df** | **p_value** | **Sig** |
| --- | --- | --- | --- | --- |
| Monocytes (fraction of CD45+) | 11.234 | 5 | 0.0469 | * |
| T Cells (fraction of CD45+) | 13.712 | 5 | 0.0175 | * |
| NKT Cells (fraction of CD45+) | 3.859 | 5 | 0.5700 | ns |
| B Cells (fraction of CD45+) | 5.574 | 5 | 0.3500 | ns |
| NK Cells (fraction of CD45+) | 6.645 | 5 | 0.2480 | ns |

## 1.5. Post-hoc Dunn Tests (BH correction)

### 1.5.1. Monocytes (fraction of CD45+)

Table 2. Dunn Test – Monocytes

| **group1** | **group2** | **statistic** | **p** | **p.adj** | **p.adj.signif** |
| --- | --- | --- | --- | --- | --- |
| Control | AD | 2.634 | 0.00844 | 0.127 | ns |
| Control | CBS | 1.532 | 0.12500 | 0.269 | ns |
| Control | FTD | 0.224 | 0.82300 | 0.909 | ns |
| Control | PSP | 2.252 | 0.02430 | 0.152 | ns |
| Control | DLB | 0.389 | 0.69700 | 0.871 | ns |
| AD | CBS | -0.910 | 0.36300 | 0.495 | ns |
| AD | FTD | -2.048 | 0.04050 | 0.152 | ns |
| AD | PSP | -0.923 | 0.35600 | 0.495 | ns |
| AD | DLB | -2.132 | 0.03300 | 0.152 | ns |
| CBS | FTD | -1.124 | 0.26100 | 0.443 | ns |
| CBS | PSP | 0.190 | 0.84900 | 0.909 | ns |
| CBS | DLB | -1.113 | 0.26600 | 0.443 | ns |
| FTD | PSP | 1.543 | 0.12300 | 0.269 | ns |
| FTD | DLB | 0.115 | 0.90900 | 0.909 | ns |
| PSP | DLB | -1.622 | 0.10500 | 0.269 | ns |

### 1.5.2. T Cells (fraction of CD45+)

Table 3. Dunn Test – T Cells

| **group1** | **group2** | **statistic** | **p** | **p.adj** | **p.adj.signif** |
| --- | --- | --- | --- | --- | --- |
| Control | AD | 1.773 | 0.07620 | 0.1900 | ns |
| Control | CBS | 1.276 | 0.20200 | 0.4330 | ns |
| Control | FTD | 0.777 | 0.43700 | 0.5960 | ns |
| Control | PSP | 0.848 | 0.39600 | 0.5960 | ns |
| Control | DLB | -1.792 | 0.07310 | 0.1900 | ns |
| AD | CBS | -0.393 | 0.69400 | 0.7440 | ns |
| AD | FTD | -0.815 | 0.41500 | 0.5960 | ns |
| AD | PSP | -1.182 | 0.23700 | 0.4450 | ns |
| AD | DLB | -3.239 | 0.00120 | 0.0180 | * |
| CBS | FTD | -0.417 | 0.67700 | 0.7440 | ns |
| CBS | PSP | -0.672 | 0.50200 | 0.6270 | ns |
| CBS | DLB | -2.730 | 0.00632 | 0.0344 | * |
| FTD | PSP | -0.154 | 0.87800 | 0.8780 | ns |
| FTD | DLB | -2.236 | 0.02530 | 0.0950 | ns |
| PSP | DLB | -2.703 | 0.00687 | 0.0344 | * |

## 1.6. CD4 Flow by Group

| **Chi_Squared** | **df** | **P_Value** |
| --- | --- | --- |
| 3.047 | 5 | 0.6927 |

## 1.7. CD4 Linear Model

| **Term** | **Estimate** | **Std_Error** | **T_Value** | **P_Value** |
| --- | --- | --- | --- | --- |
| (Intercept) | 0.465 | 0.104 | 4.450 | 0.0000 |
| groupDLB | -0.026 | 0.037 | -0.702 | 0.4837 |
| groupAD | -0.021 | 0.039 | -0.537 | 0.5921 |
| groupCBS | -0.033 | 0.040 | -0.804 | 0.4225 |
| groupFTD | 0.045 | 0.039 | 1.136 | 0.2574 |
| groupPSP | -0.024 | 0.030 | -0.796 | 0.4272 |
| Age | 0.003 | 0.001 | 2.203 | 0.0289 |
| SexM | -0.035 | 0.022 | -1.594 | 0.1129 |

## 1.8. CD8 by group

| **Chi_Squared** | **df** | **P_Value** |
| --- | --- | --- |
| 2.365 | 5 | 0.7966 |

## 1.9. CD8 Linear Model

| **Term** | **Estimate** | **Std_Error** | **T_Value** | **P_Value** |
| --- | --- | --- | --- | --- |
| (Intercept) | 0.438 | 0.099 | 4.411 | 0.0000 |
| groupDLB | 0.000 | 0.035 | -0.002 | 0.9982 |
| groupAD | 0.005 | 0.037 | 0.140 | 0.8888 |
| groupCBS | 0.010 | 0.038 | 0.271 | 0.7864 |
| groupFTD | -0.047 | 0.037 | -1.255 | 0.2111 |
| groupPSP | 0.017 | 0.029 | 0.573 | 0.5675 |
| Age | -0.003 | 0.001 | -1.874 | 0.0627 |
| SexM | 0.047 | 0.021 | 2.233 | 0.0269 |

## 1.10. CD4_CD8 ratio

| **Chi_Squared** | **df** | **P_Value** |
| --- | --- | --- |
| 2.431 | 5 | 0.7868 |

## 1.11. Th1 by group

| **Chi_Squared** | **df** | **P_Value** |
| --- | --- | --- |
| 6.687 | 5 | 0.245 |

## 1.12. Th1 Linear Model

| **Term** | **Estimate** | **Std_Error** | **T_Value** | **P_Value** |
| --- | --- | --- | --- | --- |
| (Intercept) | 0.373 | 0.085 | 4.379 | 0.0000 |
| groupAD | -0.033 | 0.032 | -1.037 | 0.3012 |
| groupCBS | 0.000 | 0.033 | 0.001 | 0.9994 |
| groupFTD | -0.046 | 0.032 | -1.427 | 0.1554 |
| groupPSP | 0.002 | 0.025 | 0.083 | 0.9338 |
| groupDLB | -0.029 | 0.030 | -0.945 | 0.3461 |
| Age | -0.001 | 0.001 | -0.727 | 0.4680 |
| SexM | -0.025 | 0.018 | -1.358 | 0.1764 |

## 1.13. Th2 by group

| **Chi_Squared** | **df** | **P_Value** |
| --- | --- | --- |
| 3.03 | 5 | 0.6953 |

## 1.14. Th2 Linear Model

| **Term** | **Estimate** | **Std_Error** | **T_Value** | **P_Value** |
| --- | --- | --- | --- | --- |
| (Intercept) | 0.229 | 0.062 | 3.723 | 0.0003 |
| groupAD | -0.010 | 0.023 | -0.433 | 0.6657 |
| groupCBS | -0.027 | 0.024 | -1.119 | 0.2648 |
| groupFTD | -0.016 | 0.023 | -0.690 | 0.4914 |
| groupPSP | -0.011 | 0.018 | -0.613 | 0.5408 |
| groupDLB | -0.003 | 0.022 | -0.149 | 0.8815 |
| Age | 0.000 | 0.001 | 0.484 | 0.6292 |
| SexM | 0.021 | 0.013 | 1.632 | 0.1046 |

## 1.15. Th17 by group

| **Chi_Squared** | **df** | **P_Value** |
| --- | --- | --- |
| 6.52 | 5 | 0.2588 |

## 1.16. Th17 Linear Model

| **Term** | **Estimate** | **Std_Error** | **T_Value** | **P_Value** |
| --- | --- | --- | --- | --- |
| (Intercept) | 0.131 | 0.050 | 2.593 | 0.0104 |
| groupAD | 0.005 | 0.019 | 0.238 | 0.8118 |
| groupCBS | -0.004 | 0.020 | -0.222 | 0.8244 |
| groupFTD | 0.016 | 0.019 | 0.870 | 0.3853 |
| groupPSP | 0.005 | 0.015 | 0.357 | 0.7215 |
| groupDLB | 0.024 | 0.018 | 1.354 | 0.1774 |
| Age | 0.001 | 0.001 | 1.539 | 0.1256 |
| SexM | 0.017 | 0.011 | 1.589 | 0.1138 |

## 1.17. Th1_17 by group

| **Chi_Squared** | **df** | **P_Value** |
| --- | --- | --- |
| 8.755 | 5 | 0.1192 |

## 1.18. Th1_17 Linear Model

| **Term** | **Estimate** | **Std_Error** | **T_Value** | **P_Value** |
| --- | --- | --- | --- | --- |
| (Intercept) | 0.267 | 0.053 | 5.052 | 0.0000 |
| groupAD | 0.039 | 0.020 | 1.953 | 0.0525 |
| groupCBS | 0.031 | 0.020 | 1.518 | 0.1309 |
| groupFTD | 0.045 | 0.020 | 2.279 | 0.0239 |
| groupPSP | 0.004 | 0.015 | 0.240 | 0.8105 |
| groupDLB | 0.008 | 0.019 | 0.406 | 0.6855 |
| Age | -0.001 | 0.001 | -0.862 | 0.3901 |
| SexM | -0.014 | 0.011 | -1.232 | 0.2195 |

## 1.19. naive by group

| **Chi_Squared** | **df** | **P_Value** |
| --- | --- | --- |
| 4.126 | 5 | 0.5314 |

## 1.20. naive Linear model

| **Term** | **Estimate** | **Std_Error** | **T_Value** | **P_Value** |
| --- | --- | --- | --- | --- |
| (Intercept) | 0.345 | 0.124 | 2.771 | 0.0062 |
| groupAD | -0.003 | 0.047 | -0.072 | 0.9426 |
| groupCBS | -0.061 | 0.048 | -1.257 | 0.2106 |
| groupFTD | -0.024 | 0.047 | -0.524 | 0.6008 |
| groupPSP | -0.066 | 0.036 | -1.820 | 0.0705 |
| groupDLB | -0.052 | 0.044 | -1.186 | 0.2373 |
| Age | 0.002 | 0.002 | 0.926 | 0.3556 |
| SexM | -0.040 | 0.026 | -1.520 | 0.1304 |

## 1.21. central memory by group

| **Chi_Squared** | **df** | **P_Value** |
| --- | --- | --- |
| 7.876 | 5 | 0.1632 |

## 1.22. central memory Linear model

| **Term** | **Estimate** | **Std_Error** | **T_Value** | **P_Value** |
| --- | --- | --- | --- | --- |
| (Intercept) | 0.153 | 0.093 | 1.649 | 0.1010 |
| groupAD | -0.055 | 0.035 | -1.557 | 0.1214 |
| groupCBS | -0.011 | 0.036 | -0.299 | 0.7652 |
| groupFTD | -0.002 | 0.035 | -0.043 | 0.9657 |
| groupPSP | -0.008 | 0.027 | -0.308 | 0.7588 |
| groupDLB | 0.062 | 0.033 | 1.867 | 0.0636 |
| Age | 0.001 | 0.001 | 0.807 | 0.4205 |
| SexM | 0.023 | 0.020 | 1.181 | 0.2391 |

## 1.23. effector memeory by group

| **Chi_Squared** | **df** | **P_Value** |
| --- | --- | --- |
| 4.952 | 5 | 0.4218 |

## 1.24. effector memory Linear model

| **Term** | **Estimate** | **Std_Error** | **T_Value** | **P_Value** |
| --- | --- | --- | --- | --- |
| (Intercept) | 0.365 | 0.116 | 3.161 | 0.0019 |
| groupAD | 0.063 | 0.043 | 1.447 | 0.1497 |
| groupCBS | 0.070 | 0.045 | 1.573 | 0.1176 |
| groupFTD | 0.029 | 0.043 | 0.669 | 0.5043 |
| groupPSP | 0.045 | 0.034 | 1.327 | 0.1861 |
| groupDLB | -0.018 | 0.041 | -0.445 | 0.6567 |
| Age | -0.001 | 0.002 | -0.812 | 0.4179 |
| SexM | 0.025 | 0.025 | 0.999 | 0.3193 |

## 1.25. EMRA by group

| **Chi_Squared** | **df** | **P_Value** |
| --- | --- | --- |
| 9.805 | 5 | 0.081 |

## 1.26. EMRA Linear model

| **Term** | **Estimate** | **Std_Error** | **T_Value** | **P_Value** |
| --- | --- | --- | --- | --- |
| (Intercept) | 0.137 | 0.054 | 2.546 | 0.0118 |
| groupAD | -0.005 | 0.020 | -0.247 | 0.8054 |
| groupCBS | 0.001 | 0.021 | 0.049 | 0.9609 |
| groupFTD | -0.003 | 0.020 | -0.150 | 0.8807 |
| groupPSP | 0.029 | 0.016 | 1.883 | 0.0614 |
| groupDLB | 0.009 | 0.019 | 0.468 | 0.6401 |
| Age | -0.001 | 0.001 | -1.802 | 0.0734 |
| SexM | -0.008 | 0.011 | -0.662 | 0.5087 |

## 1.27. CD8 naive by group

| **Chi_Squared** | **df** | **P_Value** |
| --- | --- | --- |
| 6.567 | 5 | 0.2549 |

## 1.28. CD8 naive Linear model

| **Term** | **Estimate** | **Std_Error** | **T_Value** | **P_Value** |
| --- | --- | --- | --- | --- |
| (Intercept) | 0.407 | 0.091 | 4.483 | 0.0000 |
| groupAD | -0.038 | 0.034 | -1.102 | 0.2722 |
| groupCBS | -0.012 | 0.035 | -0.335 | 0.7379 |
| groupFTD | -0.008 | 0.034 | -0.234 | 0.8155 |
| groupPSP | -0.056 | 0.026 | -2.118 | 0.0356 |
| groupDLB | 0.005 | 0.032 | 0.145 | 0.8850 |
| Age | -0.003 | 0.001 | -2.035 | 0.0434 |
| SexM | -0.060 | 0.019 | -3.119 | 0.0021 |

## 1.29. CD8 central memory by group

| **Chi_Squared** | **df** | **P_Value** |
| --- | --- | --- |
| 9.885 | 5 | 0.0786 |

## 1.30. CD8 central memory Linear model

| **Term** | **Estimate** | **Std_Error** | **T_Value** | **P_Value** |
| --- | --- | --- | --- | --- |
| (Intercept) | 0.029 | 0.050 | 0.574 | 0.5665 |
| groupAD | -0.028 | 0.019 | -1.469 | 0.1437 |
| groupCBS | 0.015 | 0.019 | 0.794 | 0.4282 |
| groupFTD | -0.009 | 0.019 | -0.463 | 0.6438 |
| groupPSP | -0.001 | 0.015 | -0.068 | 0.9461 |
| groupDLB | 0.024 | 0.018 | 1.344 | 0.1808 |
| Age | 0.000 | 0.001 | 0.566 | 0.5719 |
| SexM | -0.022 | 0.011 | -2.027 | 0.0442 |

## 1.31. CD8 effector memory by group

| **Chi_Squared** | **df** | **P_Value** |
| --- | --- | --- |
| 8.442 | 5 | 0.1335 |

## 1.32. CD8 effector memory Linear model

| **Term** | **Estimate** | **Std_Error** | **T_Value** | **P_Value** |
| --- | --- | --- | --- | --- |
| (Intercept) | 0.161 | 0.126 | 1.279 | 0.2028 |
| groupAD | 0.069 | 0.047 | 1.457 | 0.1471 |
| groupCBS | 0.058 | 0.049 | 1.193 | 0.2346 |
| groupFTD | 0.039 | 0.047 | 0.832 | 0.4068 |
| groupPSP | -0.012 | 0.037 | -0.326 | 0.7445 |
| groupDLB | -0.047 | 0.045 | -1.048 | 0.2962 |
| Age | 0.001 | 0.002 | 0.747 | 0.4560 |
| SexM | 0.031 | 0.027 | 1.141 | 0.2553 |

## 1.33. CD8 EMRA by group

| **Chi_Squared** | **df** | **P_Value** |
| --- | --- | --- |
| 7.553 | 5 | 0.1827 |

## 1.34. CD8 EMRA Linear model

| **Term** | **Estimate** | **Std_Error** | **T_Value** | **P_Value** |
| --- | --- | --- | --- | --- |
| (Intercept) | 0.404 | 0.153 | 2.635 | 0.0092 |
| groupAD | -0.004 | 0.058 | -0.064 | 0.9492 |
| groupCBS | -0.062 | 0.059 | -1.042 | 0.2987 |
| groupFTD | -0.023 | 0.057 | -0.394 | 0.6944 |
| groupPSP | 0.069 | 0.045 | 1.545 | 0.1242 |
| groupDLB | 0.018 | 0.054 | 0.335 | 0.7379 |
| Age | 0.001 | 0.002 | 0.405 | 0.6858 |
| SexM | 0.051 | 0.033 | 1.573 | 0.1177 |

## 1.35. Multinomial model

| **Outcome** | **Term** | **Estimate** | **Std_Error** | **Z_Value** | **P_Value** |
| --- | --- | --- | --- | --- | --- |
| AD | (Intercept) | -3.476 | 2.936 | -1.184 | 0.2365 |
| AD | Age | 0.039 | 0.041 | 0.940 | 0.3471 |
| AD | SexM | -0.144 | 0.604 | -0.238 | 0.8116 |
| AD | PC1 | -0.697 | 0.380 | -1.835 | 0.0665 |
| AD | PC2 | -0.292 | 0.686 | -0.425 | 0.6706 |
| AD | PC3 | -0.107 | 0.305 | -0.353 | 0.7243 |
| AD | PC4 | 0.593 | 0.321 | 1.848 | 0.0646 |
| AD | PC5 | 0.051 | 0.441 | 0.117 | 0.9072 |
| CBS | (Intercept) | -1.209 | 2.891 | -0.418 | 0.6758 |
| CBS | Age | 0.018 | 0.041 | 0.436 | 0.6628 |
| CBS | SexM | -1.346 | 0.627 | -2.145 | 0.0320 |
| CBS | PC1 | -0.183 | 0.327 | -0.558 | 0.5771 |
| CBS | PC2 | 0.191 | 0.511 | 0.374 | 0.7087 |
| CBS | PC3 | 0.271 | 0.302 | 0.897 | 0.3696 |
| CBS | PC4 | 0.326 | 0.328 | 0.995 | 0.3195 |
| CBS | PC5 | 0.305 | 0.315 | 0.967 | 0.3334 |
| FTD | (Intercept) | 0.796 | 2.752 | 0.289 | 0.7723 |
| FTD | Age | -0.020 | 0.040 | -0.515 | 0.6067 |
| FTD | SexM | 0.055 | 0.606 | 0.091 | 0.9274 |
| FTD | PC1 | 0.179 | 0.307 | 0.581 | 0.5615 |
| FTD | PC2 | 0.216 | 0.556 | 0.388 | 0.6983 |
| FTD | PC3 | -0.110 | 0.305 | -0.361 | 0.7179 |
| FTD | PC4 | 0.613 | 0.309 | 1.982 | 0.0475 |
| FTD | PC5 | -0.007 | 0.402 | -0.018 | 0.9860 |
| PSP | (Intercept) | -3.097 | 2.268 | -1.366 | 0.1720 |
| PSP | Age | 0.056 | 0.032 | 1.760 | 0.0783 |
| PSP | SexM | -0.555 | 0.462 | -1.201 | 0.2299 |
| PSP | PC1 | -0.041 | 0.240 | -0.171 | 0.8643 |
| PSP | PC2 | 0.698 | 0.404 | 1.728 | 0.0840 |
| PSP | PC3 | 0.246 | 0.224 | 1.098 | 0.2723 |
| PSP | PC4 | 0.253 | 0.257 | 0.982 | 0.3263 |
| PSP | PC5 | -0.002 | 0.294 | -0.006 | 0.9951 |
| DLB | (Intercept) | -7.916 | 2.995 | -2.643 | 0.0082 |
| DLB | Age | 0.092 | 0.041 | 2.279 | 0.0227 |
| DLB | SexM | 1.355 | 0.679 | 1.996 | 0.0460 |
| DLB | PC1 | 0.375 | 0.271 | 1.386 | 0.1658 |
| DLB | PC2 | 0.513 | 0.442 | 1.161 | 0.2457 |
| DLB | PC3 | 0.123 | 0.282 | 0.437 | 0.6623 |
| DLB | PC4 | -0.086 | 0.326 | -0.263 | 0.7928 |
| DLB | PC5 | 0.231 | 0.340 | 0.680 | 0.4963 |

## 1.36. FTD vs control logistic model

| **Term** | **Estimate** | **Std_Error** | **Z_Value** | **P_Value** |
| --- | --- | --- | --- | --- |
| (Intercept) | 0.313 | 2.816 | 0.111 | 0.9115 |
| Age | -0.013 | 0.040 | -0.335 | 0.7377 |
| SexM | 0.069 | 0.590 | 0.116 | 0.9073 |

## 1.37. FTD model with PC4

| **Term** | **Estimate** | **Std_Error** | **Z_Value** | **P_Value** |
| --- | --- | --- | --- | --- |
| (Intercept) | 0.376 | 2.946 | 0.128 | 0.8983 |
| Age | -0.015 | 0.042 | -0.353 | 0.7243 |
| SexM | 0.088 | 0.612 | 0.143 | 0.8862 |
| PC4 | 0.639 | 0.333 | 1.920 | 0.0549 |

## 1.38. Model comparison PC4

**Output**

Analysis of Deviance Table

Model 1: group ~ Age + Sex

Model 2: group ~ Age + Sex + PC4

Resid. Df Resid. Dev Df Deviance Pr(>Chi)

1	52	71.965

2	51	67.558 1	4.4077 0.03578 *

---

Signif. codes: 0 ‘***’ 0.001 ‘**’ 0.01 ‘*’ 0.05 ‘.’ 0.1 ‘ ’ 1

## 1.39. Model comparison NFL

**Output**

Analysis of Deviance Table

Model 1: group ~ Age + Sex

Model 2: group ~ Age + Sex + NFL

Resid. Df Resid. Dev Df Deviance Pr(>Chi)

1	37	50.137

2	36	10.865 1	39.272 3.687e-10 ***

---

Signif. codes: 0 ‘***’ 0.001 ‘**’ 0.01 ‘*’ 0.05 ‘.’ 0.1 ‘ ’ 1

## 1.40. Model addition of PC4

**Output**

Analysis of Deviance Table

Model 1: group ~ Age + Sex + NFL

Model 2: group ~ Age + Sex + NFL + PC4

Resid. Df Resid. Dev Df Deviance Pr(>Chi)

1	36	10.865

2	35	10.557 1 0.30854	0.5786

## 1.41. FTD vs other disease logistic model

| **Term** | **Estimate** | **Std_Error** | **Z_Value** | **P_Value** |
| --- | --- | --- | --- | --- |
| (Intercept) | -2.006 | 2.243 | -0.895 | 0.3710 |
| Age | 0.058 | 0.032 | 1.806 | 0.0709 |
| SexM | -0.438 | 0.510 | -0.858 | 0.3910 |

## 1.42. FTD vs other disease with PC4

| **Term** | **Estimate** | **Std_Error** | **Z_Value** | **P_Value** |
| --- | --- | --- | --- | --- |
| (Intercept) | -2.194 | 2.259 | -0.971 | 0.3314 |
| Age | 0.061 | 0.033 | 1.888 | 0.0590 |
| SexM | -0.393 | 0.514 | -0.764 | 0.4451 |
| PC4 | -0.315 | 0.218 | -1.442 | 0.1492 |

## 1.43. Model comparison PC4 vs others

**Output**

Analysis of Deviance Table

Model 1: FTD_group ~ Age + Sex

Model 2: FTD_group ~ Age + Sex + PC4

Resid. Df Resid. Dev Df Deviance Pr(>Chi)

1	140	111.75

2	139	109.75 1	1.9944	0.1579

## 1.44. FTD vs other disease base

| **Term** | **Estimate** | **Std_Error** | **Z_Value** | **P_Value** |
| --- | --- | --- | --- | --- |
| (Intercept) | -3.328 | 2.597 | -1.281 | 0.2001 |
| Age | 0.079 | 0.039 | 2.018 | 0.0436 |
| SexM | -0.472 | 0.625 | -0.755 | 0.4501 |

## 1.45. FTF vs other disease NFL

| **Term** | **Estimate** | **Std_Error** | **Z_Value** | **P_Value** |
| --- | --- | --- | --- | --- |
| (Intercept) | -3.445 | 2.671 | -1.290 | 0.1972 |
| Age | 0.095 | 0.041 | 2.301 | 0.0214 |
| SexM | -0.487 | 0.648 | -0.752 | 0.4519 |
| NFL | -0.021 | 0.010 | -2.154 | 0.0312 |

## 1.46. Model comparison NFL vs others

**Output**

Analysis of Deviance Table

Model 1: FTD_group ~ Age + Sex

Model 2: FTD_group ~ Age + Sex + NFL

Resid. Df Resid. Dev Df Deviance Pr(>Chi)

1	95	75.828

2	94	71.304 1	4.5234 0.03343 *

---

Signif. codes: 0 ‘***’ 0.001 ‘**’ 0.01 ‘*’ 0.05 ‘.’ 0.1 ‘ ’ 1

## 1.47. Survival model

| **Term** | **HR** | **Estimate** | **Std_Error** | **Z_Value** | **P_Value** |
| --- | --- | --- | --- | --- | --- |
| PC1 | 0.741 | -0.300 | 0.151 | -1.988 | 0.0468 |
| PC2 | 1.127 | 0.120 | 0.122 | 0.985 | 0.3248 |
| PC3 | 0.627 | -0.466 | 0.177 | -2.632 | 0.0085 |
| PC4 | 0.843 | -0.171 | 0.193 | -0.885 | 0.3764 |
| PC5 | 1.160 | 0.148 | 0.128 | 1.155 | 0.2480 |
| groupCBS | 1.738 | 0.553 | 1.024 | 0.539 | 0.5896 |
| groupPSP | 12.446 | 2.521 | 0.774 | 3.256 | 0.0011 |
| groupFTD | 5.019 | 1.613 | 0.802 | 2.012 | 0.0443 |
| groupDLB | 3.792 | 1.333 | 0.792 | 1.684 | 0.0922 |
| ACER_total | 0.990 | -0.010 | 0.008 | -1.183 | 0.2367 |
| SexM | 0.786 | -0.240 | 0.340 | -0.707 | 0.4794 |
| year | 0.874 | -0.134 | 0.082 | -1.643 | 0.1004 |
| Age | 1.059 | 0.058 | 0.024 | 2.450 | 0.0143 |

## 1.48. Survival model NFL

| **Term** | **HR** | **Estimate** | **Std_Error** | **Z_Value** | **P_Value** |
| --- | --- | --- | --- | --- | --- |
| PC1 | 0.873 | -0.136 | 0.192 | -0.708 | 0.4786 |
| PC3 | 0.573 | -0.557 | 0.211 | -2.638 | 0.0083 |
| Age | 1.017 | 0.017 | 0.028 | 0.616 | 0.5380 |
| ACER_total | 1.001 | 0.001 | 0.011 | 0.097 | 0.9228 |
| SexM | 0.686 | -0.377 | 0.424 | -0.889 | 0.3742 |
| groupCBS | 1.899 | 0.641 | 1.453 | 0.441 | 0.6590 |
| groupPSP | 22.658 | 3.121 | 1.073 | 2.908 | 0.0036 |
| groupFTD | 6.636 | 1.893 | 1.128 | 1.677 | 0.0935 |
| groupDLB | 6.558 | 1.881 | 1.078 | 1.744 | 0.0811 |
| NFL | 1.014 | 0.014 | 0.008 | 1.727 | 0.0842 |
| pTau217 | 2.306 | 0.835 | 0.460 | 1.814 | 0.0696 |

# 2. Supplementary figures

**Supplementary figure 1:** Gating strategy. Note that for CD4+ ‘Th’ cells, as provisionally indicated on the bivariate plots here, naïve cells were removed post-data acquisition (e.g., Th1 cells would be defined as the counts from the CD4+CXCR3+CCR6- gate minus counts from the derivative CD45RA+CCR7+ gate). In working with these data, we found this gating strategy improved interrater reliability in a subset of samples.  Thus, all data presented on Th cells in the main paper are memory cell populations, consistent with the differentiation of T helper subsets after naïve cell exposure to antigen.

**Supplementary figure 2:**  A. Batch effect of CD8+ and CD4+ cells across the collected samples. B. Batch effect

of CD8+ and CD4+ cells across collected control samples.

**Supplementary figure 3:** Gating strategy for the additional broad immunophenotyping panel performed to test whether differences in T-cell populations were related to differences in other major cell populations.

**Supplementary figure 4:** Associations of CD3+ cells as fraction of PBMCs with Abeta42/40 ratio, pTau217, pTau231, NfL and GFAP in patients with DLB. Plots show the residuals of log plasma biomarker and CD3+ cell as a fraction of total PBMCs from a linear model including age and sex.

**Supplementary figure 5:** Associations of CD8+ naïve cells as fraction of CD8+ cells with Abeta42/40 ratio, pTau217, pTau231, NfL and GFAP in patients with PSP. Plots show the residuals of log plasma biomarker and CD8+ naïve cells as a fraction of total CD8 cells from a linear model including age and sex.

**Supplementary figure 6:** Associations of Th1/17 cells as fraction of CD4+ memory cells with Abeta42/40 ratio, pTau217, and pTau231 in patients with FTD. Plots show the residuals of log plasma biomarker and Th1/17 cells as a fraction of total CD4+ memory cells from a linear model including age and sex.

**Supplementary figure 7:** Scree plot and cumulative variance plot for the principal components analysis.

**Supplementary figure 8:** ROC plots for logistic regression models comparing the base model (including age and sex only) with the addition of principal component 4 individual scores (left plots) or plasma NfL (right plots) in FTD compared to controls (top row) or compared to other neurodegenerative diagnosis (bottom plots). The AUC for the base model is plotted in red, and for the full model plotted in blue.

# 3. Supplementary tables

| **Cell population** | **Rho** | **P-value** | **Sig.** |
| --- | --- | --- | --- |
| CD3+ | -0.133 | 0.445 |  |
| CD4+ | 0.043 | 0.806 |  |
| CD8+ | -0.042 | 0.811 |  |
| Th1-like | 0.202 | 0.243 |  |
| Th2-like | 0.288 | 0.093 |  |
| Th17-like | -0.239 | 0.166 |  |
| Th1_17-like | -0.278 | 0.106 |  |
| CD4 naïve | 0.002 | 0.989 |  |
| CD4 CM | -0.163 | 0.351 |  |
| CD4 EM | 0.073 | 0.676 |  |
| CD4 EMRA | 0.395 | 0.019 | * |
| CD8 naïve | 0.052 | 0.769 |  |
| CD8 CM | -0.030 | 0.866 |  |
| CD8 EM | 0.084 | 0.632 |  |
| CD8 EMRA | 0.038 | 0.827 |  |
| ** p < 0.05  \|  Spearman correlation with flow_date_2* | | | |

**Supplementary table 1:** Spearman correlation coefficients between the populations included in the analysis and the date of sampling in controls. Only CD4 EMRA cells, as a proportion of CD4 cells showed a significant correlation.

| Variable | Median | IQR (Q1-Q3) |
| --- | --- | --- |
| time_cells_singlets_live | 387,208.0 | 246404 - 532848.5 |
| time_cells_singlets_live_cd3 | 197,963.5 | 113800.25 - 257557 |
| time_cells_singlets_live_cd3_cd4 | 123,670.5 | 71899.5 - 172377.5 |
| time_cells_singlets_live_cd3_cd4_th1 | 23,532.5 | 13783 - 34399.75 |
| time_cells_singlets_live_cd3_cd4_th1_cm | 4,974.5 | 2205 - 8008.75 |
| time_cells_singlets_live_cd3_cd4_th1_em | 10,006.0 | 4406 - 17660 |
| time_cells_singlets_live_cd3_cd4_th1_emra | 1,172.0 | 581.25 - 2487.5 |
| time_cells_singlets_live_cd3_cd4_th17 | 15,803.5 | 9583.75 - 24988 |
| time_cells_singlets_live_cd3_cd4_th17_cm | 5,924.0 | 2716.5 - 10206.25 |
| time_cells_singlets_live_cd3_cd4_th17_em | 6,924.5 | 3716.75 - 12336 |
| time_cells_singlets_live_cd3_cd4_th17_emra | 173.0 | 69.25 - 404.5 |
| time_cells_singlets_live_cd3_cd4_th1_17 | 16,004.0 | 8162 - 24249.75 |
| time_cells_singlets_live_cd3_cd4_th1_17_cm | 3,503.0 | 1413.25 - 6787.5 |
| time_cells_singlets_live_cd3_cd4_th1_17_em | 10,123.0 | 4671.75 - 17390.5 |
| time_cells_singlets_live_cd3_cd4_th1_17_emra | 219.5 | 98.25 - 522.25 |
| time_cells_singlets_live_cd3_cd4_th2 | 58,210.5 | 31193.5 - 90443.5 |
| time_cells_singlets_live_cd3_cd4_th2_cm | 9,217.0 | 4402.75 - 14178.5 |
| time_cells_singlets_live_cd3_cd4_th2_em | 4,772.5 | 2349.25 - 9176.25 |
| time_cells_singlets_live_cd3_cd4_th2_emra | 762.5 | 336.25 - 1800 |
| time_cells_singlets_live_cd3_cd8 | 46,830.0 | 23940.25 - 74368.75 |
| time_cells_singlets_live_cd3_cd8_cm | 1,075.5 | 337.5 - 3261.75 |
| time_cells_singlets_live_cd3_cd8_em | 11,232.0 | 3681 - 22624.75 |
| time_cells_singlets_live_cd3_cd8_emra | 19,026.5 | 8543.25 - 47958.75 |
| time_cells_singlets_live_cd3_cd8_naive | 5,249.5 | 2620.75 - 9883.5 |

**Supplementary table 2:** Cell numbers in each gate: median + interquartile range for each population across all patient samples

| Biomarker | Control | DLB | AD | CBS | FTD | PSP | Kruskal-Wallis Test (χ², p-value) |
| --- | --- | --- | --- | --- | --- | --- | --- |
| pTau217 | 0.32 ± 0.22 | 0.68 ± 0.4 | 0.91 ± 0.38 | 0.54 ± 0.47 | 0.41 ± 0.22 | 0.48 ± 0.47 | 38.05, p=< 0.001 |
| pTau231 | 6.07 ± 2.28 | 11.03 ± 7.04 | 11.37 ± 5.4 | 8.8 ± 4 | 8.06 ± 5.09 | 9.12 ± 4.51 | 23.5, p=< 0.001 |
| GFAP | 105.71 ± 55.08 | 173.26 ± 91.95 | 204.09 ± 106.3 | 206.94 ± 92.99 | 167.25 ± 101.59 | 170.22 ± 127.68 | 18.89, p=0.002 |
| NFL | 18.08 ± 6.76 | 38.08 ± 17.83 | 33.06 ± 13.87 | 61.78 ± 45.22 | 58.72 ± 25.51 | 46.22 ± 24.79 | 50.43, p=< 0.001 |

**Supplementary table 3:** Plasma biomarker results split by group, all measurements in pg/mL.

|  | **Overall**   N = 178^1^ | **No plasma biomarkers**   N = 54^1^ | **Plasma biomarkers**   N = 124^1^ | **p-value**^2^ |
| --- | --- | --- | --- | --- |
| **Age** | 71 (65, 76) | 71 (66, 78) | 71 (65, 76) | 0.10 |
| **Sex** |  |  |  | >0.9 |
| F | 75 (42%) | 23 (43%) | 52 (42%) |  |
| M | 103 (58%) | 31 (57%) | 72 (58%) |  |
| **Group** |  |  |  | 0.014 |
| Control | 35 (20%) | 9 (17%) | 26 (21%) |  |
| AD | 20 (11%) | 2 (3.7%) | 18 (15%) |  |
| CBS | 19 (11%) | 6 (11%) | 13 (10%) |  |
| FTD | 20 (11%) | 6 (11%) | 14 (11%) |  |
| PSP | 58 (33%) | 27 (50%) | 31 (25%) |  |
| DLB | 26 (15%) | 4 (7.4%) | 22 (18%) |  |
| **ACE-R** | 83 (69, 92) | 82 (70, 92) | 83 (69, 93) | 0.8 |
| ^1^Median (Q1, Q3); n (%) | | | | |
| ^2^Wilcoxon rank sum test; Pearson's Chi-squared test | | | | |

**Supplementary table 4:** Demographic differences between those with plasma biomarkers and those without plasma biomarkers.

| Population name | Markers |
| --- | --- |
| CD4_Naive | CD3+, CD4+, CXCR3-, CCR6-, CD45RA+, CCR7+ |
| CD4 Th1 CM | CD3+, CD4+, CXCR3+, CCR6-, CD45RA-, CCR7+ |
| CD4 Th2 CM | CD3+, CD4+, CXCR3-, CCR6-, CD45RA-, CCR7+ |
| CD4 Th17 CM | CD3+, CD4+, CXCR3-, CCR6+, CD45RA-, CCR7+ |
| CD4 Th1_17 CM | CD3+, CD4+, CXCR3+, CCR6+, CD45RA-, CCR7+ |
| CD4 Th1 EM | CD3+, CD4+, CXCR3+, CCR6-, CD45RA-, CCR7- |
| CD4 Th2 EM | CD3+, CD4+, CXCR3-, CCR6-, CD45RA-, CCR7- |
| CD4 Th17 EM | CD3+, CD4+, CXCR3-, CCR6+, CD45RA-, CCR7- |
| CD4 Th1_17 EM | CD3+, CD4+, CXCR3+, CCR6+, CD45RA-, CCR7- |
| CD4 Th1 EMRA | CD3+, CD4+, CXCR3+, CCR6-, CD45RA+, CCR7- |
| CD4 Th2 EMRA | CD3+, CD4+, CXCR3-, CCR6-, CD45RA+, CCR7- |
| CD4 Th17 EMRA | CD3+, CD4+, CXCR3-, CCR6+, CD45RA+, CCR7- |
| CD4 Th1_17 EMRA | CD3+, CD4+, CXCR3+, CCR6+, CD45RA+, CCR7- |
| CD8_Naive | CD3+, CD8+, CD45RA+, CCR7+ |
| CD8_CM | CD3+, CD8+, CD45RA-, CCR7+ |
| CD8_EM | CD3+, CD8+, CD45RA-, CCR7- |
| CD8_EMRA | CD3+, CD8+, CD45RA+, CCR7- |

**Supplementary table 5:** Table showing the cell populations included in the PCA
